# Changes in Whey Proteome between Mediterranean and Murrah Buffalo Colostrum and Mature Milk Reflect Their Pharmaceutical and Medicinal Value

**DOI:** 10.3390/molecules27051575

**Published:** 2022-02-27

**Authors:** Mahmoud Abdel-Hamid, Pan Yang, Islam Mostafa, Ali Osman, Ehab Romeih, Yongxin Yang, Zizhen Huang, Awad A. Awad, Ling Li

**Affiliations:** 1 Guangxi Buffalo Research Institute, Chinese Academy of Agricultural Sciences, Nanning 530001, China; mahmoud.mohamed@agr.cu.edu.eg (M.A.-H.); hnyangpan2008@163.com (P.Y.); hzz90302@163.com (Z.H.); 2 Dairy Science Department, Faculty of Agriculture, Cairo University, Giza 12613, Egypt; ehab.romeih@staff.cu.edu.eg (E.R.); awad.awad@agr.cu.edu.eg (A.A.A.); 3 Department of Pharmacognosy, Faculty of Pharmacy, Zagazig University, Zagazig 44519, Egypt; i_m_elbaz@zu.edu.eg; 4 Biochemistry Department, Faculty of Agriculture, Zagazig University, Zagazig 44511, Egypt; aomokhalil82@gmail.com; 5 College of Food Science and Engineering, Qingdao Agricultural University, Qingdao 266109, China; yangyx1216@126.com

**Keywords:** Mediterranean buffalo, Murrah buffalo, colostrum, mature milk, proteomics, LC-MS/MS

## Abstract

Milk represents an integrated meal for newborns; its whey protein is rich in many health beneficial components and proteins. The current study aimed to investigate the differences between colostrum and mature milk from Mediterranean and Murrah buffaloes using labeled proteomics and bioinformatics tools. In the current work, LC-MS/MS analysis led to identification of 780 proteins from which 638 were shared among three independent TMT experiments. The significantly changed proteins between the studied types were analyzed using gene ontology enrichment and KEGG pathways, and their interactions were generated using STRING database. Results indicated that immunological, muscular development and function, blood coagulation, heme related, neuronal, translation, metabolic process, and binding proteins were the main terms. Overall, colostrum showed higher levels of immunoglobulins, myosins, actin, neurofascin, syntaxins, thyroglobulins, and RNA-binding proteins, reflecting its importance in the development and activity of immunological, muscular, cardiac, neuronal, and thyroid systems, while lactoferrin and ferritin were increased in mature milk, highlighting its role in iron storage and hemoglobin formation.

## 1. Introduction

Buffalo is a member of bovine animals that is classified into several species from which Asian buffalo (*Bubalus bubalis*) is the most domesticated one. It is further classified into river and swamp buffalo subspecies [1]. River buffalo is often called water buffalo and has a higher milk yield than swamp buffalo, which are reared for meat production. Moreover, milk production is affected by the genetic background of the animal. The river buffaloes are the most numerous of the species and include Murrah, Jafarabadi, Surti, Mehsana, Nili Ravi, Egyptian, and Mediterranean breeds (about 80% of the world’s buffalo population). They are raised in a region extending from India in the east to Italy in the west, including several countries like Pakistan, Iraq, Syria, Egypt, Greece, and Bulgaria [1,2]. In addition, Carabao and Buffalypso are the common buffalo breeds used for meat production [2]. 

Milk represents a mammalian biological fluid that has great importance attributed to its nutritional and functional benefits for humans [3]. These benefits can range from supplying the body with essential metabolites and growth factors such as proteins, carbohydrates, lipids, vitamins, and calcium that contribute to the development and activity of cardiovascular, immunological, and neuronal systems, as well as gut microbiota. Whey proteins represent the soluble proteins of milk and constitute about 20% of the total milk proteins. Whey proteins such as α-lactalbumin, β-lactoglobulin, bovine serum albumin, immunoglobulins, lactoferrin, and lactoperoxidase are potentially contributing to the nutritional and biological importance of milk. Moreover, whey proteins possess several pharmaceutical interests including antioxidant, anti-inflammatory, anticancer, and antimicrobial activities [4]. Whey proteins show different compositions between different buffalo species with some specific proteomes to each one [5]. Several studies investigated the proteome of milk’s whey and milk fat globule membrane (MFGM) proteins from many milk-producing species based on different proteomics approaches such as gel, label-free, and label (iTRAQ and TMT)-based proteomics techniques [6]. Previous studies reported the differences between colostrum and milk in Holstein cows using proteomics approaches. These studies revealed differences in immune, lactotransferrin, gastro-intestinal tract maturation, and blood clotting-related proteins between the two types [7,8,9]. Similar results were observed in Holstein and Jersey breeds [10]. The composition of milk from cows and buffaloes is different [11], and the environment of animal growth could be of great importance in milk production and composition [12].

Best to our knowledge, few studies were focused on the proteome of buffalo colostrum and mature milk and their biological values. Therefore, the present study aimed to define the main differences and nutritional and biological values of whey proteins of colostrum and mature milk from Mediterranean and Murrah buffalo breeds using labeled proteomics tools.

## 2. Results

### 2.1. Compositional Analysis of the Investigated Milk Types

Analyses of the total protein, fat, lactose, and solids contents of the different milk types were performed, and statistical significance between the differences was considered at *p*-value < 0.05. The results showed that colostrum milk contains higher protein content compared to that of mature milk, whereas mature milk had higher lactose content in both Mediterranean and Murrah buffaloes. On the other hand, no significant differences were observed between Mediterranean and Murrah mature milk (*p* > 0.05) or their colostrum types (Table 1).

### 2.2. Whey Proteome of Mediterranean and Murrah Colostrum and Mature Milk

The LC-MS/MS analysis of TMT tagged peptides of whey proteins of investigated samples resulted in the identification of 780 proteins from Mediterranean and Murrah colostrum and mature milk in three independent experiments. Of these proteins, 638 were sharing all three runs and were consequently available for quantification. Statistical analysis of the quantified proteins showed that 139 and 25 proteins had increased and decreased colostrum levels, respectively, compared to that of mature milk of Mediterranean. Similarly, 134 proteins increased and 57 proteins decreased in colostrum compared to that of mature milk of Murrah buffalo. Moreover, 41 proteins were increased and 57 were decreased in Mediterranean colostrum relative to Murrah colostrum. Further, 53 proteins increased and 104 proteins decreased in Mediterranean mature milk compared to that of Murrah mature milk (Table S1, Supplementary file 1).

#### 2.2.1. Common Protein Changes between the Investigated Milk Types 

A total of seven proteins exhibited significant level changes between the different types of milk. They include unconventional myosin-Ic, cadherin-13, adenylate kinase isoenzyme 1, mitochondrial isocitrate dehydrogenase (NADP), odorant-binding protein, 40S ribosomal protein S19, heterogeneous nuclear ribonucleoprotein A/B, and 60S ribosomal protein L27a. These proteins are related to several biological processes such as intracellular movements, calcium-binding, ATP/AMP phosphorylation, metabolism and energy production, odorant- and small molecule-binding, RNA-binding, and translation (Table S1).

#### 2.2.2. Proteins That Showed Highest and Lowest Production Levels between the Studied Milk Types 

Starting with the colostrum and mature milks of Mediterranean, results showed that Ig-like domain-containing protein (G3MZE0), thyroglobulin, Fc-gamma-RII-D, and IGK protein increased by 11.75-, 7.73-, 5.7-, and 4-fold, respectively, in colostrum compared to that of mature milk. On the other hand, heart fatty acid-binding protein, prosaposin, and platelet glycoprotein 4 showed 0.31-, 0.37-, and 0.38-fold decrease, respectively, in colostrum in comparison to those of mature milk. By comparing changes in colostrum relative to mature milk in Murrah, the colostrum exhibited increases in Ig-like domain-containing protein (G3MZE0) (FC = 5.25), Fc-gamma-RII-D (FC = 4.76), and Ig-like domain-containing protein (A0A3Q1LL87) (FC = 4.55) and decreases in desmin (FC = 0.2), lactoperoxidase (FC = 0.3), lipoprotein lipase (FC = 0.3), heart fatty acid-binding protein (FC = 0.33), dolichol-diphosphooligosaccharide-protein glycosyltransferase 48 kDa subunit (FC = 0.33), alpha-lactalbumin (FC = 0.35), beta-lactoglobulin (FC = 0.35), prosaposin (FC = 0.36), C-C motif chemokine (FC = 0.37), alpha-lactalbumin (FC = 0.37), platelet glycoprotein 4 (FC = 0.38), beta-1,4-galactosyltransferase 1 (FC = 0.39), and isocitrate dehydrogenase 1 (FC = 0.39).

With respect to colostrum of Mediterranean and colostrum of Murrah, thyroglobulin, myomesin (M-protein) 2, 165kDa, globin B1, and desmin were the proteins with the highest increases, with 4.49-, 4.36-, 4.19-, and 4.1-fold increases, respectively. Meanwhile, heterogeneous nuclear ribonucleoprotein A/B, Y-box-binding protein 1, and reticulon were the proteins that decreased the most, with 0.25-, 0.29-, and 0.3-fold decreases, respectively. Concerning the differences between Mediterranean mature milk and Murrah mature milk, the proteins with the highest fold changes in Mediterranean compared to the that of Murrah were adenosylhomocysteinase 3 (FC =7.96), calcium-dependent secretion activator (FC = 4.95), and V-type proton ATPase subunit G (FC = 4.13), while those with the lowest fold change in Mediterranean relative to the Murrah were reticulon (FC = 0.16), dolichol-diphosphooligosaccharide-protein glycosyltransferase 48 kDa subunit (FC = 0.24), histone H2A type 1 (FC = 0.33), PC4, and SFRS1-interacting protein (FC = 0.34), odorant-binding protein-like (FC = 0.34), serpin H1 (FC = 0.35), and testin (FC = 0.37). The changed proteins cover a broad spectrum of biological processes including immunological response, muscles building and activity, blood integrity, neurotransmission and development, thyroid activity, and growth and translation process. Details regarding the functions of these proteins are presented in Supplementary file 1.

### 2.3. Parallel Reaction Monitoring Confirmed the Accuracy of TMT Labelling and Relative Quantitation

**Figure 1:**
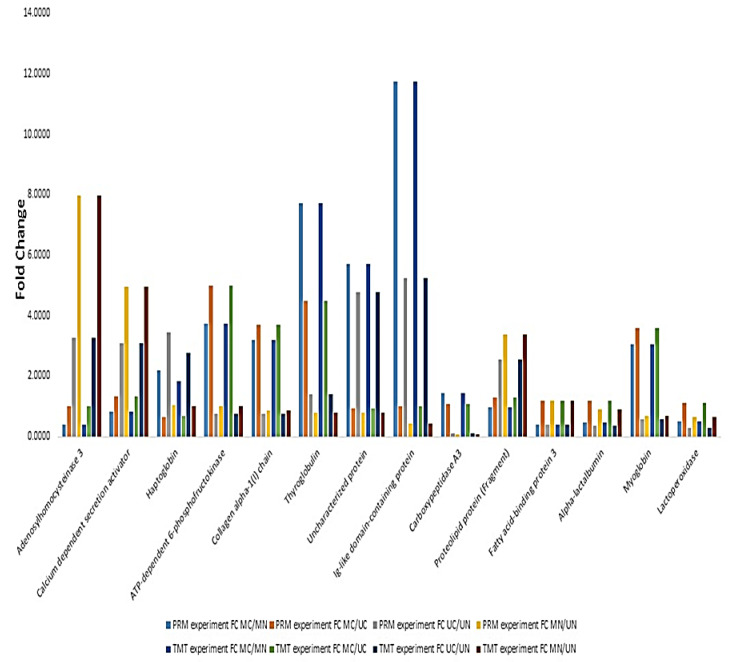
Relationship between relative quantitation values of selected proteins using parallel reaction monitoring (PRM) and TMT labeling. MC: Mediterranean colostrum; MN: Mediterranean mature milk; UC: Murrah colostrum; UN: Murrah mature milk.

PRM experiment was performed for the quantification of 14 selected proteins. Among these, Ig-like domain-containing protein was the most changed protein in colostrum and mature milk for both Mediterranean and Murrah, experiencing about 11.75- and 5.25-fold increases in colostrum groups, respectively. ATP-dependent 6-phosphofructokinase was the most increased protein in the colostrum of Mediterranean relative to that of Murrah (5-fold increase), while adenosylhomocysteinase 3 was the most increased one in Mediterranean mature milk compared to that of the Murrah mature milk (7.96-fold increase). Quantification values are provided in Supplementary file 1. The results showed a high similarity between TMT and PRM experiments on the level of selected proteins (Figure 1).

### 2.4. Principle Component Analysis and Heatmap Clustering

**Figure 2:**
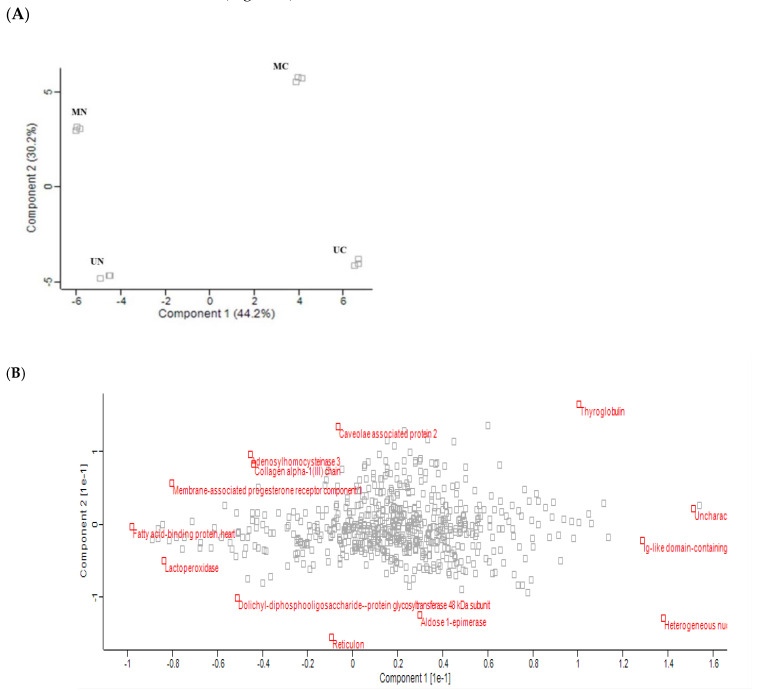
Principle component analysis of quantified proteins in three TMT-independent experiments from Mediterranean colostrum (MC), Mediterranean mature milk (MN), Murrah colostrum (UC), and Murrah mature milk (UN). (**A**) scores plot; percentage of variation explained by each component is shown between brackets. (**B**) loading plot.

**Figure 3:**
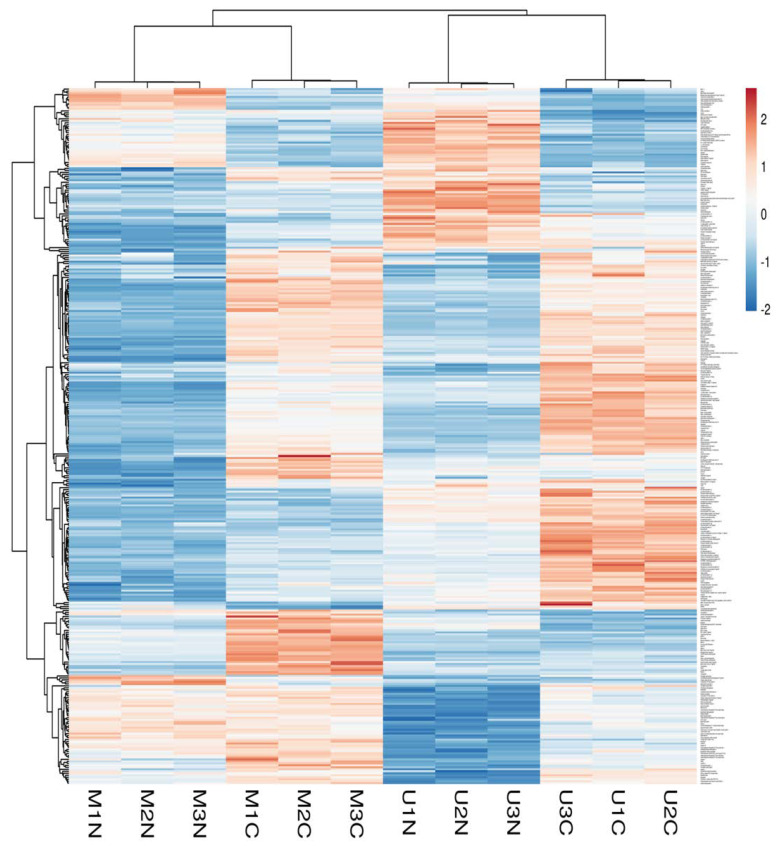
Heatmap of differentially changed proteins at a *p*-value < 0.01 and cutoff points >1.5 and <0.667 for fold changes of increased and decreased proteins, respectively, in three TMT independent experiments from Mediterranean colostrum (MC), Mediterranean mature milk (MN), Murrah colostrum (UC), and Murrah mature milk (UN). Blue and red colors indicate lowest and highest protein fold changes.

Principle component analysis (PCA) of the quantified proteins showed distinct categorization of Mediterranean and Murrah colostrum and mature milk into four categories as revealed from the score plot (Figure 2A). The loading plot shows the related protein variables such as thyroglobulin, 40S ribosomal protein, lactoperoxidase, fatty acid-binding protein, adenosylhomocysteinase, and collagen alpha-1 (III) chain (Figure 2B). Heat mapping of the differentially expressed proteins confirmed this categorization with little similarities between each pair of colostrum and mature milk clusters. Moreover, comparing colostrum and mature milk of each type revealed no similarity between their clusters (Figure 3).

### 2.5. Gene Ontology and Pathway Analyses of Differentially Expressed Proteins

The differences between Mediterranean colostrum milk and its mature milk-covered 35 GO biological process (BP) terms, 23 GO molecular function (MN) terms, and 20 GO cellular compartmentalization (CC) terms. Medically interesting ones from the BP group include innate immune response, collagen fibril organization, and muscle contraction, and biologically important MF terms are poly(A) RNA binding, calcium ion binding, protein binding, nucleotide binding, actin binding, and receptor binding. KEGG pathway analysis showed that the differentially expressed proteins are linked to 12 pathways, and those related to coagulation, immunity, and infection, focal adhesion and translation are of significant interest (Supplementary file 2). 

By comparing Murrah colostrum milk to its mature one, we found that BP GO enrichment included 48 terms and translation, and of particular interest were in utero embryonic development, angiogenesis, cell adhesion, protein transport, platelet aggregation, and endocytosis. The MF GO enrichment included 20 terms, and poly(A) RNA-binding, protein-binding, structural constituent of ribosome, receptor-binding, and serine-type endopeptidase inhibitor activity are the most important. Concerning CC, 29 GO terms were enriched. Pathway analysis using KEGG revealed 13 records, and the major ones were translation, complement and coagulation cascades, immunity, carbon metabolism, endocytosis, focal adhesion, and synaptic vesicle cycle (Supplementary file 2).

The changed proteins between Mediterranean and Murrah colostrum involved 16 BP GO terms from which translation and muscle contraction were the richest. Among 11 MF GO terms, poly(A) RNA-binding, structural constituent of ribosome, actin-binding, nucleotide-binding, and RNA-binding showed the highest counts, and there were 22 CC GO terms. These proteins were included in four KEGG pathways that are related to translation and immunity (Supplementary file 2). 

As to the differentially changed proteins between Mediterranean and Murrah mature milk, 45 BP, 24 MF, and 40 CC GO terms were recorded. Translation, protein folding, protein stabilization, and negative regulation of endopeptidase activity were the richest counts from the BP group, and poly(A) RNA binding, structural constituent of ribosome, protein folding, unfolded protein-binding, and RNA-binding were in the top counts of MF group. The KEGG pathway analysis of this group revealed 21 pathways of which translation, immunity, protein processing in the endoplasmic reticulum, and focal adhesion were the richest pathways (Supplementary file 2).

### 2.6. Interaction Networks of the Changed Proteins Using the STRING Software

**Figure 4:**
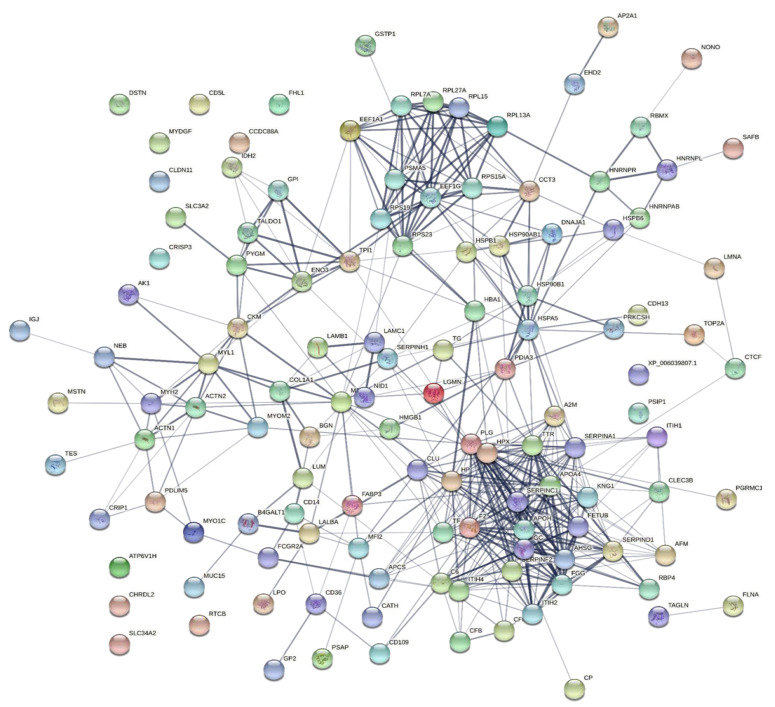
Map showing relationship of differentially expressed proteins between Mediterranean colostrum and mature milk using STRING in confidence view. Interaction score: medium confidence, 0.4; the interactions between nodes rely on coexpression, co-occurrence, gene fusion, neighborhood, published results, and text-mining information. Connection strength is directly proportional to edge thickness. Significant differences at *p*-value < 0.01 and cut-off points >1.5 and <0.667 for fold changes of increased and decreased proteins, respectively, in three TMT-independent experiments were considered for this analysis. Description of nodes is provided in Supplementary file 2.

**Figure 5:**
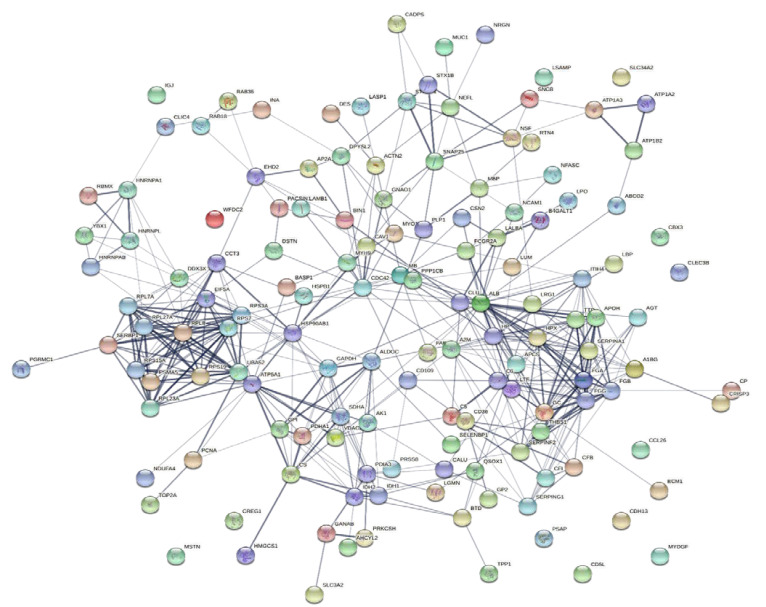
Map showing relationship of differentially expressed proteins between Murrah colostrum and mature milk using STRING in confidence view. Interaction score: medium confidence 0.4. Interactions between nodes rely on coexpression, co-occurrence, gene fusion, neighborhood, published results, and text-mining information. Connection strength is directly proportional to edge thickness. Significant differences at *p*-value < 0.01, and cut-off points were >1.5 and <0.667 for fold changes of increased and decreased proteins, respectively, in three TMT-independent experiments were considered for this analysis. Description of nodes is provided in Supplementary file 2.

**Figure 6:**
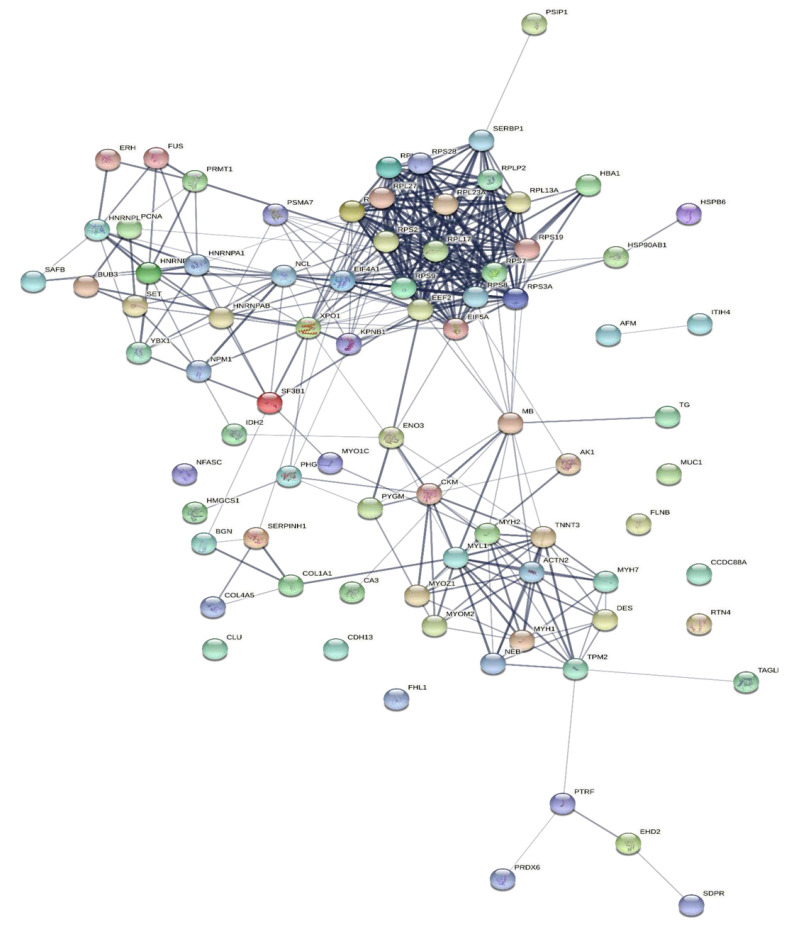
Map showing relationship of differentially expressed proteins between colostrums of Mediterranean and Murrah buffaloes using STRING in confidence view. Interaction score: medium confidence 0.4. Interactions between nodes rely on coexpression, co-occurrence, gene fusion, neighborhood, published results, and text-mining information. Connection strength is directly proportional to edge thickness. Significant differences at *p*-value < 0.01 and cut-off points were >1.5 and <0.667 for fold changes of increased and decreased proteins, respectively, in three TMT-independent experiments were considered for this analysis. Description of nodes is provided in Supplementary file 2.

**Figure 7:**
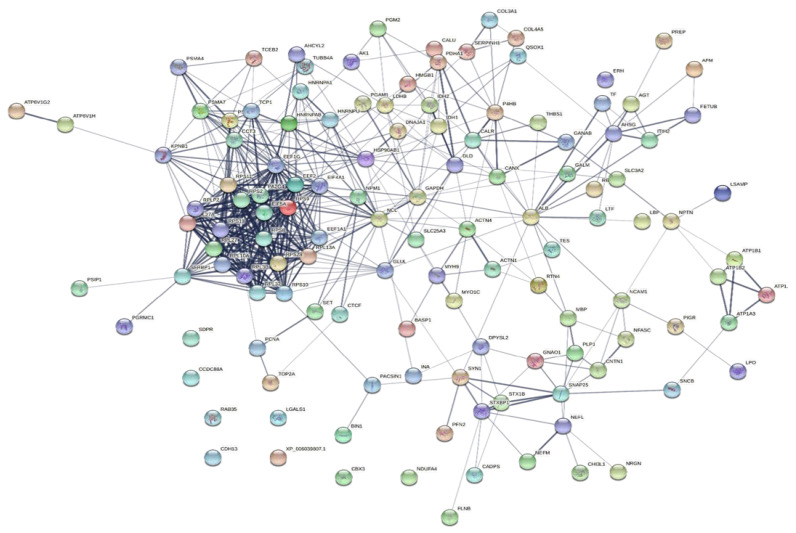
Map showing relationship of differentially expressed proteins between mature milk of Mediterranean and Murrah buffaloes using STRING in confidence view Interaction score: medium confidence, 0.4. Interactions between nodes rely on coexpression, co-occurrence, gene fusion, neighborhood, published results, and text-mining information. Connection strength is directly proportional to edge thickness. Significant differences at *p*-value < 0.01 and cut-off points were >1.5 and <0.667 for fold changes of increased and decreased proteins, respectively, in three TMT-independent experiments were considered for this analysis. Description of nodes is provided in Supplementary file 2.

To explore the correlations between the changed proteins in each group comparison, the STRING software was used to generate networking interaction maps. STRING succeeded in mapping 124, 81, 126, and 141 proteins from MC/MN (Figure 4), UC/UN (Figure 5), MC/UC (Figure 6), and MN/UN (Figure 7) groups, respectively, using *Bubalus bubalis* species. Moreover, 113, 71, 117, and 128 of the mapped proteins were able to interconnect to form the networks in MC/MN, MC/UC, MN/UN, and UC/UN, respectively. The maps revealed the strong functional relationship between the differentially expressed proteins, which is directly proportional to the thickness of the edges connecting the protein nodes. STRING analysis indicated several hubs of immune-related proteins, such as serpin family members (SERPIN) and complement component C6, blood-related proteins including fibrinogen chains (FGA, B and/or G) and haptoglobin (HP), and translation proteins (ribosomal proteins) in both colostrum types, relative to their mature milk (Figure 4 and Figure 5). Regarding the relationship between colostrum peers, the central hubs were clear for translation (ribosomal proteins) and muscular and calcium-related proteins, such as myosins (MYH) and alpha-actinin-2 (ACTN2) (Figure 6). Both mature milk types showed a compact cluster for translation proteins (ribosomal proteins, elongation factors (EEF), and translation initiation factors (EIF)), as well as serum albumin (ALB) which is involved in the binding process (Figure 7).

## 3. Discussion

### 3.1. Protein Changes of Pharmaceutical and Medical Interest

#### 3.1.1. Proteins Related to Immunological Development

Newborns have low immunity [13]. This requires their food to be rich in immunological factors, and the current study is in agreement regarding the importance of colostrum milk as a rich source for immune-related proteins [9,14,15]. Previous reports revealing this fact include increased expression of osteopontin, haptoglobin, milk amyloid A, and gelsolin in colostrum milk [8], and increased immunoglobulins levels in colostrum in the first two days of life [9]. Elevated levels of immunoglobulin were also reported in Ovine whey colostrum [16]. In the current study, there is evidence for the increased immunological proteins in Mediterranean and Murrah colostrum. These include Ig-like domain-containing proteins A0A3Q1LL87, G3MZE0, G3N148, and G3N342, which potentiate B cell receptor signaling pathway and antigen-binding [17]; alpha-1 acid glycoprotein, which regulates immune response [18]; beta-2-glycoprotein 1, which is involved in the functionality of innate immunity [19]; immunoglobulin J chain, which participates in IgA and immunoglobulin formation and receptor-bindings [20]; complement component C6, which makes punctures in the plasma membrane of target cells [21], and serum amyloid P-component, which was found to regulate macrophages and neutrophil adhesion [22]. Monocyte differentiation antigen CD14 is supposed to enhance the immune response to bacterial lipopolysaccharides [23], biglycan is involved in the innate immune and inflammatory responses [24], and complement factor H balances immune response and participates in the clearance of dead and microbial cells [25], which were increased in Mediterranean colostrum. Besides, the antigen processing protein beta-2-microglobulin was increased in Murrah colostrum [26]. 

#### 3.1.2. Proteins Related to Muscular and Connective Tissues Development and Activity

The process of muscle contraction is dependent on calcium concentration as well as actin, troponin, and myosin proteins [27,28]. Moreover, the previous factors can play an important role in muscle development and growth [29], reflecting the valuable role of milk that is rich in these proteins for neonate growth. Mediterranean colostrum showed higher content of alpha-actinin-2, MYL1, and EH-domain containing 2 than that of its mature milk and colostrum of Murrah. Alpha-actinin-2 acts on actin and calcium bindings, and its activation leads to muscle contraction [30]; whereas, the other two proteins that are involved in calcium-binding were higher in the mature Murrah milk than in its colostrum. Tropomyosin beta chain and troponin T fast skeletal muscle type are other protein examples that regulate cardiac and skeletal striated muscle contraction. Furthermore, they are of great value in cardiac function and contraction [31,32,33], and both proteins has higher increases in Mediterranean colostrum milk than in Murrah colostrum. Cadherin-13 is a calcium ion binding protein, and it plays a crucial role in skeletal muscle differentiation and signaling transduction to muscles besides their neurodevelopmental effect [34,35,36]. Colostrum milk showed increased values of this protein compared to that of the mature ones of both Mediterranean and Murrah buffaloes; moreover, its level was higher in Mediterranean than the Murrah in general. With respect to myosin proteins, myosin-1, -2, and -7 increased in Mediterranean colostrum over Murrah colostrum. Myosin heavy chain 9 showed decreased levels in both Murrah colostrum and Mediterranean mature milk relative to Murrah mature milk, while myosin heavy chain 2x and myosin heavy chain (M1R8X4) were increased in Mediterranean colostrum compared to that of its mature milk and Murrah colostrum one. These results demonstrate the beneficial impact of Mediterranean colostrum on muscular system development and activity. Another interesting finding related to this part is the elevated concentration of vitamin D-binding protein in both colostrum types, as this protein participates in vitamin D storage and transport, intestinal calcium absorption, and consequently, bone-building [37].

Additionally, collagens are responsible for the formation of connective tissues, ligands, and supporting organs [38]. In this work, many collagen-related proteins were increased in specific milk types more than others. For instance, collagen type IV alpha 5 chain was increased in both Mediterranean milk types compared to that of their corresponding Murrah milk types. Collagen alpha-1(I) chain showed higher levels in colostrum Mediterranean relative to its mature milk, as well as to the Murrah colostrum. However, collagen alpha-1(III) chain was increased in mature Mediterranean than the mature Murrah. Biglycan is another protein related to this class via its role in collagen fibers assembly, formation of bones, and integrity of muscles [24]. Its level in Mediterranean colostrum was higher than that of mature Mediterranean and Murrah colostrum types. Lumican, a protein responsible for the organization and binding of collagen fibers [39], showed higher concentrations in colostrum than corresponding mature ones. Serpin H1 is a collagen-binding protein and is required for collagen synthesis [40]. Its level was higher in Mediterranean colostrum compared to that of Mediterranean mature milk, while it was lower in Murrah colostrum and Mediterranean mature compared to that of Murrah mature. From the above observations, it is obvious that Mediterranean colostrum and mature milk are more beneficial for the building of connective tissues and ligaments in comparison to that of the Murrah milk types. 

#### 3.1.3. Proteins Related to Fibrinolytic Activity and Blood Formation and Integrity

Dupont [41] reported the presence of fibrinolytic enzymes in colostrum and mature milk. Zhang et al. reported the relation of coagulation proteins to different milk types including colostrum [9]. Our results demonstrated that colostrum milk has more anticoagulant properties relative to mature milk. Examples that prove this postulation include antithrombin-III (thrombin inhibitor) and SERPIND1 protein, which were higher in colostrum Mediterranean milk than the mature one, and these proteins act as thrombin inhibitors [42]. Similarly, the previous immune-related protein beta-2-glycoprotein 1 exerts anticlotting activity via binding to heparin [19]. Another interesting protein that supports this finding is the fibrin dissolving protein plasminogen [43], which showed an elevated level in colostrum Mediterranean compared to that of mature Mediterranean milk. The decreased concentration of platelet glycoprotein 4 in both colostrum types compared to their corresponding mature milk types represents more evidence in this regard.

As to blood formation, iron is known to be a key element in several biological processes; in particular, blood formation, as well as the formation of oxygen transport proteins [44]. Lactotransferrin is an iron-binding protein that regulates the binding and transport of ferric ions; moreover, it can suppress nasopharyngeal carcinoma, besides its role in innate immunity [45]. Lactotransferrin showed higher content in mature Murrah milk than that of its colostrum or the mature milk of Mediterranean. Lactoferrin, another iron-binding protein, exhibited a higher level in Murrah mature milk than that of its colostrum. Lactoferrin attracts high attention nowadays due to its antimicrobial activity, anti-SASR-CoV-2 action, and other potential bioactivities [46]. Furthermore, globin domain-containing protein, globin B1, and myoglobin proteins showed higher contents in Mediterranean colostrum than that of its mature milk and the colostrum of Murrah; furthermore, the second was higher in mature Mediterranean than the mature Murrah, while the last one (myoglobin) was higher in the mature Murrah than its colostrum. By contrast, hemoglobin subunit alpha-1 was higher in Murrah colostrum than mature one, and hemoglobin subunit alpha-2 was higher in mature Mediterranean than the corresponding Murrah one. These enzymes are essential for the process of transporting oxygen from lungs to peripheral tissues and oxidation/reduction reactions [44,47]. Hemoglobin subunit beta is another oxygen transport protein [48] that exhibited elevated levels in Mediterranean mature milk than its colostrum and the mature milk of Murrah, while its level in Murrah colostrum was higher than that of Murrah mature milk. The iron-binding and transport proteins melanotransferrin and serotransferrin were also higher in Mediterranean colostrum than in its mature milk [49,50]. Hemopexin is another protein that is related to this group, and it is responsible for heme-binding and transport to the liver for its breakdown and iron liberation [51]. Its level was higher in both colostrum types compared to both mature milk types. Similarly, the hemoglobin binding protein haptoglobin, which possesses an antioxidant and antibacterial activities [51], was higher in both colostrum types. In contrast, Ferritin concentration was lower in colostrum than in mature Murrah milk. This enzyme is required for the storage of iron insoluble form [52]. From the above, we may conclude that each milk type can potentially contribute to hemoglobin formation, iron-binding, and transfer by utilizing different kinds of heme-related proteins. 

#### 3.1.4. Proteins Related to Neuronal System Development and Activity

Milk plays an important role in neuronal development and neurotransmission [53]. The present study showed that milk type can highly influence the neuronal development and transmission. Evidence concerning this finding demonstrated that mature Mediterranean milk exhibited a superior effect compared to that of mature Murrah milk. However, Murrah colostrum milk is superior to its mature milk. This evidence includes increased concentrations of: (a) neurofascin (participant in axon guidance, neurotransmission, and peripheral nervous system development) [54], (b) dihydropyrimidinase-related protein 2 (related to axon guidance and brain development) [55], (c) neural cell adhesion molecule 1 (involved in neuronal adhesion and growth) [56], (d) neurofilament light polypeptide (maintains neuronal caliber, size, and shape) [57], (e) neurogranin (messenger and participant in synaptic development) [58], (f) alpha-internexin (neuron morphogenesis), (g) beta-synuclein (required for neuronal plasticity) [59], (h) syntaxin-1B and syntaxin-binding protein 1 (involved in synaptic vesicles docking and transmission) [33], (i) syntaxins (are required for neuronal development) [60], (j) synaptosomal-associated protein 25 (neurotransmitter release regulator) [61], (k) PC4 and SFRS1-interacting protein (participants in neurogenesis and differentiation of neuroepithelial stem cells) [62]. The first protein (neurofascin) also exhibited higher content in colostrum Mediterranean than that of colostrum Murrah milk. Other protein examples that showed increased contents in mature Mediterranean over the mature Murrah are SYN1 protein (secretion of neurotransmitters) [63], neurofilament medium polypeptide (maintain neuronal caliber) [64], and neuroplastin (axon guidance and cell adhesion) [65].

#### 3.1.5. Proteins Related to Thyroid Hormones and Growth Development

Thyroid hormones are of great value in regulating metabolic processes in the body [66]. An increased production of some thyroid-related proteins was indicated in colostrum milk, especially the Mediterranean type, demonstrating its importance in the growth developmental process. Thyroglobulin is one of the proteins that showed higher level in colostrum Mediterranean compared to that of its mature milk and the Murrah colostrum milk This protein is involved in hormonal activity and biosynthetic processes [67]. Another example that showed elevated levels in both colostrum over the mature ones is transthyretin, which acts as a thyroid-binding protein and transports thyroxine from the bloodstream to the brain and other organs [68]. Alpha-2-HS-glycoprotein is a multifunctional protein that was increased in colostrum Mediterranean and decreased in mature Mediterranean. It plays a role in thyroid-binding besides its endocytosis and lymphocytes stimulation activities [69]. 

### 3.2. Protein Changes Related to the Growth Process and Electrolyte Balance

Sodium/potassium ATPase subunits represent a model for ion pumps that regulate multiple biological processes such as membrane resting, electrical connectivity of muscles and nerves, osmotic balance, and signaling molecules with an impact on the development and growth process [70]. Many members of this class showed expression changes between investigated milk types. Sodium/potassium-transporting ATPase subunit beta (Q3ZCH8) is essential for regulating ions transfer through membranes [71], and it exhibited higher concentrations in Mediterranean mature milk than Murrah one. Sodium/potassium-transporting ATPase subunit alpha plays a role in potassium transmembrane transport. In addition to ATP and metal ion-bindings [71], sodium/potassium-transporting ATPase subunit alpha-2, sodium/potassium-transporting ATPase subunit beta (L0CMU6), and sodium/potassium-transporting ATPase subunit beta-2 (Q28030) all enhance ATP hydrolysis via sodium/potassium exchange through membranes. These proteins were higher in both Murrah colostrum and Mediterranean mature milk over the Murrah mature one.

### 3.3. Protein Changes Related to the Translation Process and Organs Development

The binding and processing of RNA are of great importance in the early growth stage for the development of skeletal muscles and other body organs [72]. In the present study, overproduction of some of the enzymes responsible for these processes in colostrum milk was noted. RNA-binding motif protein of X chromosome, heterogeneous nuclear ribonucleoprotein A/B, heterogeneous nuclear ribonucleoprotein L, 60S ribosomal protein L5, ribosomal protein L23a, ribosomal protein S19, and 60S ribosomal protein L7a constitute examples for this class.

## 4. Materials and Methods

### 4.1. Collection of Milk and Preparation of Milk Whey 

Milk samples were collected from six Mediterranean and six Murrah buffaloes at two lactation stages: colostrum (0–3 days) and mature milk (two months), from the Guangxi Buffalo Research Institute farm (Nanning City, Guangxi Province, China). All buffalos were aged between five and seven years. The animals were healthy, housed in the same farm, fed with the same basal diet, and had a somatic cell in a range of 1.2 to 2.1×10^5^ cells/mL. Samples from individual buffaloes were pooled into three samples based on their lactation stage. This resulted in three replicates for each type of milk with a total of four groups; namely, Mediterranean colostrum (MC), Mediterranean mature milk (MN), Murrah colostrum (UC), and Murrah mature milk (UN). Milk composition, i.e., protein, fat, lactose, and total solid contents, was measured using a MilkoScan analyzer (FT120, FOSS, Hillerød, Denmark). 

Milk samples were defatted by centrifugation at 3000× *g* for 15 min at 4 °C. Whey was obtained from the skim milk by adjusting the pH to 4.6 using 33% acetic acid followed by 3.3 M sodium acetate. To remove casein, the mixture was centrifuged at 14,000× *g* for 30 min 20 °C. 

### 4.2. Digestion of Proteins and Labeling with Tandem Mass Tag

Starting with 500 µg from the supernatant of each sample, SDS-PAGE was performed and caseins were cut off, followed by in-gel digestion for remaining whey proteins as illustrated below.

A total of 800 μL of 0.1M NH_4_HCO_3_/30%ACN was added to each sample to destain the blue color. Then, 40 μL of 100 mM DTT and 360 μL of 100 mM NH_4_HCO_3_ were added to each sample and incubated at 56 °C for 30 min for protein reduction. Each sample was alkylated in a dark place at room temperature for 20 min using 120 μL of 200 mM iodoacetamide (IAA) in presence of 280 μL of 100 mM NH_4_HCO_3_. After alkylation, the gel was washed with 100 μL of 100 mM NH_4_HCO_3_ and dehydrated with 100 μL of ACN, and then freeze-dried. To digest the proteins, trypsin in 50 mM NH_4_HCO_3_ was added to each sample and placed in a refrigerator at 4 °C for about 30 min. Next, 50 mM NH_4_HCO_3_ buffer was added to the protein digestion mixture and kept overnight at 37 °C. The tryptic peptides were collected and transferred to a new centrifuge tube, and then 100 μL of 60% ACN/0.1%TFA (trifluoroacetic acid) were added, the tubes were rubber blocked and sonicated for 15 min, and then the supernatants were collected again and lyophilized. The dried peptides were desalted on a C18 StageTip column and lyophilized to be ready for labeling.

Equal amounts of peptides from each sample were labeled with tandem mass tag (TMT) reagents, as described in the manufacturer’s instructions (Thermo Fisher Scientific, Waltham, MA, USA). Three independent experiments were carried out, and in each experiment, MC, MN, UN, and UC samples were labeled with 126, 127, 128, and 129 reagents, respectively. After tagging, the labeled samples of each experiment were pooled together into one tube, dried, and fractionated into 10 fractions using reverse-phase chromatography as previously described [6]. Each fraction was dried and dissolved in 0.1% FA for LC-MS analysis.

### 4.3. LC-MS/MS Identification and Quantification of TMT Labeled Peptides

The analysis was carried out as previously described by Li et al. [6] and Mostafa et al. [73] with some modifications. Each fraction was subjected to chromatographic separation using the Easy nLC 1200 chromatography system (Thermo Scientific, Waltham, MA, USA) with a nanoliter flow rate. The mobile phase included two buffers: buffer A was 0.1% (*v/v*) formic acid in MilliQ water, and buffer B was 0.1% (*v/v*) formic acid in 95% acetonitrile in MilliQ water solution. Samples were loaded onto C-18 trap column (100 μm, 20 mm, 5 μm) and separated on C-18 reversed-phase column (75 μm, 150 mm, 3 μm), and the separation run was at a flow rate of 300 NL/min. The mobile phase gradient was 2–7% (*v/v*) buffer B within 3 min, 7–35% (*v/v*) buffer B within 45 min, 35–90% (*v/v*) buffer B within 5 min, and finally, isocratic of 90% (*v/v*) buffer B for 7 min. 

MS data were acquired using a data-dependent method that dynamically chooses the 20 most abundant precursor ions from the survey scan (Full MS) (mass range: 350–1800 *m/z*) for HCD fragmentation (MS/MS) in 60 min. The full MS scan was acquired with the following parameters: resolution: 60,000 at *m/z* 200; automatic gain control (AGC) target: 3e6, maximum IT: 50 ms. The MS/MS scans parameters were set as: resolution: 15,000 at *m/z* 200; AGC target: 1e5; maximum IT: 50 ms; isolation window: 1.6 *m/z*; and normalized collision energy: 32.

Raw files were subjected to MaxQuant 1.6.0.16 (http://www.maxquant.org) and searched against housed bovine and buffalo databases (downloaded from UniProt, https://www.uniprot.org accessed on 27 March 2020). An initial search was set for a precursor mass tolerance of 20 ppm. The search followed an enzymatic cleavage of Trypsin/P and allowed maximal two missed cleavage sites and a mass tolerance of 4.5 ppm for fragment ions. The modification set was as following: fixed modifications: Carbamidomethyl©, TMT6plex (K), TMT6plex (N-term); variable modifications: oxidation (M) and acetyl (Protein N-term). A minimum of six amino acids per peptide, ≥1 unique peptides were required per protein. For peptide and protein identification, the false discovery rate (FDR) was set to 1%. TMT reporter ion intensity was used for quantification.

Sharing proteins between the three experiments were considered for the determination of changes between the different milk types, and significant changes were considered at cut-off points >1.5 and <0.667 and at a *p*-value < 0.01 [74,75]. 

### 4.4. Parallel Reaction Monitoring (PRM) Analysis

PRM was carried out using Q Exactive HF-X mass spectrometer (Thermo Scientific, Waltham, MA, USA) to confirm the protein expression levels [76]. Briefly, 14 proteins were selected according to the results of the TMT approach, and their peptides were prepared as described by TMT protocol. After their digestion with trypsin, the peptides were desalted using C18 stage tips and then subjected to reversed-phase chromatography using an Easy nLC-1200 system (Thermo Scientific). The separation runs for one hour with a flow rate of 300 NL/min. The mobile phase included two buffers; buffer A was 0.1% (*v/v*) formic acid in MilliQ water, and buffer B was 0.1% (*v/v*) formic acid in 95% acetonitrile in MilliQ water solution. The mobile phase gradient was 2–8% (*v/v*) buffer B within 2 min, 8–40% (*v/v*) buffer B within 40 min, 40–55% (*v/v*) buffer B within 8 min, 55–100% (*v/v*) buffer B for 1 min, and finally, 100% (*v/v*) buffer B for 9 min. Collision energy, charge state, and retention times were optimized for the most significantly regulated peptides based on high-intensity and high-confidence unique peptides from each target protein. The Q Exactive HF-Xmass spectrometer (Thermo Scientific) was set to the following parameters: positive ionization mode, full MS1 scan was acquired with the resolution of 60,000 (at 200 *m/z*), 3.0 × 106 for automatic gain control (AGC), and 250 ms for maximum ion injection times. Full MS scans were followed by 20 PRM scans at 30,000 resolution (at *m/z* 200) with 3.0 × 106 AGC and 200 ms as maximum injection time. The targeted peptides were isolated with a window of 2Th and fragmented at normalized collision energy of 28 in higher energy dissociation (HCD) collision cell. Skyline (MacCoss Lab, University of Washington) was used for calculating signal intensities for individual peptide sequences [77].

RawMeat (version 2.1, VAST Scientific, www.vastscientific.com accessed on 27 March 2020) was used for extracting base peak intensity for each sample’s average from the full scan acquisition. For samples normalization, a factor was calculated as fN= the average base peak intensity of a sample/the median of average base peak intensities for all samples. This factor was multiplied by the area under the curve (AUC) of each transition from the sample. Following normalization, the summation of AUCs of different transitions was calculated to get AUCs at the peptide level. Relative protein abundance was defined as the intensity of the selected peptide.

### 4.5. Gene Ontology Enrichment and Bioinformatics Analysis

Functional annotation of the differentially expressed proteins was enriched using the David Bioinformatics Resources (https://david.ncifcrf.gov/home.jsp accessed on 23 August 2021) on the levels of biological process, molecular function, and cellular components. The pathways of the investigated proteins were identified based on the KEGG database (https://www.genome.jp/kegg/pathway.html accessed on 23 August 2021). The interactions between the significantly changed proteins were determined using the STRING database (https://string-db.org accessed on 23 August 2021) in the confidence view, the interactions between nodes rely on coexpression, co-occurrence, gene fusion, neighborhood, published results, and text-mining information. The functions of the significant changes proteins were retrieved from the Uniprot database (https://www.uniprot.org/ accessed on 17 August 2021).

### 4.6. Statistical and Multivariate Analysis

For compositional milk analysis, results were calculated as means of six buffaloes ± standard deviation, and significant difference was considered at *p* < 0.05 using a *t*-test.

As for protein changes from TMT proteomics analysis, the significantly changed proteins between each pair of the investigated groups were calculated using a *t*-test at a *p*-value < 0.01 and cut-off points >1.5 and <0.667 for increased and decreased proteins, respectively. For protein analysis using the PRM experiment, the ANOVA test was used to compare investigated proteins quantitation between different groups at a *p*-value < 0.01. Principle component analysis (PCA) of quantified proteins was performed using Perseus software (http://www.perseus-framework.org accessed on 16 December 2021), and heat mapping (Hierarchical clustering) of differentially expressed proteins was carried out with the aid of ClustVis (https://biit.cs.ut.ee/clustvis/ accessed on 16 December 2021).

## 5. Conclusions

The present study investigated Mediterranean and Murrah colostrum and mature milk whey proteins using a proteomics approach. LC-MS/MS analysis of the tagged peptides from trypsinized proteins resulted in the identification of 780 proteins. Analysis of the differentially expressed proteins using different bioinformatics tools such as Uniprot, GO terms, KEGG, and STRING revealed that the milk stage is of great influence on supplying the body with the necessary immunological and growth factors. It showed that the Mediterranean type is of great interest, as it is rich in proteins necessary for different biological processes and development such as immune response, muscle and collagen development and activity, blood integrity, neuronal integrity, and thyroid activity. These results confirm the importance of colostrum as the main meal for neonates and infants in the early developmental stages. On the other hand, mature milk may be essential for anemic infants due to its high content of lactoferrin and ferritin. Further investigations are required to prove these hypotheses using in vivo studies.

## Figures and Tables

**Table 1:** Chemical composition of different milk types.

Buffalo Type *	Protein	Fat	Lactose	Total Solid
Mediterranean Colostrum	9.40 ± 1.7 ^A^	9.47 ± 0.7 ^A^	3.38 ± 0.6 ^B^	24.49 ± 2.1 ^A^
Mediterranean Mature	4.42 ± 0.3 ^B^	7.50 ± 0.9 ^AB^	5.15 ± 0.4 ^A^	18.12 ± 1.0 ^B^
Murrah Colostrum	11.80 ± 3.0 ^A^	6.58 ± 1.8 ^AB^	3.11 ± 0.4 ^B^	22.79 ± 5.4 ^AB^
Murrah Mature	4.82 ± 0.3 ^B^	5.49 ± 4.0 ^B^	5.30 ± 0.4 ^A^	17.98 ± 1.9 ^B^

* Results are means of six buffaloes ± standard deviation. Different superscripts letters in the same column are significantly different (*p* ˂ 0.05).

## Data Availability

Not applicable.

## Supplementary Materials

The following supporting information can be downloaded online. Table S1: List of differentially expressed proteins and their fold changes between Mediterranean colostrum (MC), Mediterranean mature milk (MN), Murrah colostrum (UC), and Murrah mature milk (UN); Supplementary file 1: Raw data, LC-MS/MS results, proteins’ fold changes and functions; Supplementary file 2: Gene Ontology, KEGG, and STRING analyses.
