# Supplementary material for: Changes in Whey Proteome between Mediterranean and Murrah Buffalo Colostrum and Mature Milk Reflect Their Pharmaceutical and Medicinal Value
Source: Molecules. 2022 Feb 27;27(5):1575. doi: 10.3390/molecules27051575 (PMC8912021; doi:10.3390/molecules27051575)
Supplement: Supplementary file 1 [file molecules-27-01575-s001.zip › molecules-1588243-supplementary/Table S1.pdf]

Table S1. List of differentially expressed proteins and their fold changes between Murrah coloctrum (UC), Murrah mature milk (UN), Mediterranean colostrum (MC) and Mediterranean mature milk (MN).

Protein names	Uniprot ID	Gene names	FC MC/MN*	FC UC/UN*	FC MC/UC*	FC MN/UN*
Beta-2-microglobulin	A0A023JPW3	B2M		1.5750271		
Solute carrier family 3 member 2	A0A089FXT1	SLC3A2	2.3034424	1.5689947		0.6532476
SERPIN domain-containing protein	A0A0A0MPA0	LOC784932		1.6421333		
Heat shock protein 90 variant AB1 (Fragment)	A0A0A0YM85	H			0.6108003	
Complement component 5	A0A0F6T485	C5		0.5657955		
RNA-binding protein FUS	Q28009	FUS			0.5966035	
Serum albumin	P02769	ALB		0.619217		0.6513466
Heat shock protein beta-6 (Fragment)	Q148F8	HspB6	2.0258899		1.9219659	
Heat shock protein family B member 1 variant 1	A0A097PIG9	H	1.9098968			
Monocyte differentiation antigen CD14	Q1PBD0	CD14	2.2868117			
Ribosomal protein L15 (Fragment)	A0A345W857	RPL15	1.679436			
Lactotransferrin	Q0PGA3	LTF		0.561709		0.5381037
Calnexin	A7Z066	CANX				0.5341211
Chordin like 2	Q2KJ20	CHRDL2	2.3392974			
Nebulin	A0A3Q1LGQ8	NEB	2.0442472		2.2764266	
Angiotensinogen	Q3SZH5	AGT		2.1733576		1.6854106
Inter-alpha-trypsin inhibitor heavy chain H2	P56651	ITIH2	2.2910546			0.6642949
Ig-like domain-containing protein	A0A3Q1LL87		3.4714772	4.5489315	0.6404759	
Myelin proteolipid protein	P04116	PLP1		2.693641		2.9024163
Uncharacterized protein	A0A3Q1LPG0		2.9023069	1.9058074		
Nucleolin	E1B8K6	NCL			0.5296125	0.6155745
Inter-alpha-trypsin inhibitor heavy chain H1	Q0VCM5	ITIH1	1.7828654			
Ig-like domain-containing protein	G3MZE0		11.749575	5.2479694		0.4489158
Myosin-1	Q9BE40	MYH1			1.9560156	
Uncharacterized protein	A0A3Q1LZA2	LSAMP		1.6187464		1.6942946
Glucosidase II alpha subunit	A6QNJ8	GANAB		0.5768307		0.6178262
Filamin B	E1BKX7	FLNB			0.5862683	0.5881281
Glycoprotein 2	Q0IIA4	GP2	2.1814933	1.8331133		
26S proteasome regulatory subunit 6B	Q3T030	PSMC4				0.5556311
Complement factor I	Q32PI4	CFI	1.7841367	2.1195136		
Uncharacterized protein	A0A3Q1M3L6		3.8314938	3.038899		
Apolipoprotein A-IV	Q32PJ2	APOA4	1.9884086			
Unconventional myosin-Ic	Q27966	MYO1C	2.2934617	0.6390053	1.8788027	0.5234728
Ubiquitin-like domain-containing protein	P63048	LOC101902760	1.5650144	1.5127451		
C-C motif chemokine	A0A3Q1M5V9	CCL26		0.3684857		
Alpha-actinin-2	Q3ZC55	ACTN2	1.9259162	0.5560627	2.5335011	
Guanine nucleotide-binding protein G(o) subunit alpha	P08239	GNAO1		1.6595627		2.1843583
CD109 molecule	F1MPE1	CD109	0.6274452	0.407134		
Inter-alpha-trypsin inhibitor heavy chain H4	Q3T052	ITIH4	1.6519493	2.5723908	0.6661658	
Sodium/potassium-transporting ATPase subunit beta	Q3ZCH8	ATP1B1				2.0634364
Serine protease 8	Q08DU0	PRSS8		1.6476921		
Neurofascin	G3MYY6	NFASC		2.1527497	1.6232703	2.7407806
Ribosomal_S13_N domain-containing protein	Q56JX8			1.6778055	0.5141074	0.5912112
Methanethiol oxidase	Q2KJ32	SELENBP1		0.5585719		
Pyruvate dehydrogenase E1 component subunit alpha	Q2T9Y3	PDHA1		1.754009		1.9723023
Serine/threonine-protein phosphatase	Q3SWW9	PPP1CB		1.6346074		
Destrin	Q5E9D5	DSTN	1.6971197	1.9740535		
60S ribosomal protein L13a	Q7M2Y2	RPL13A	1.5184812		0.5812514	0.5420643
Dihydropyrimidinase-related protein 2	O02675	DPYSL2		1.6879144		1.9413196
Uncharacterized protein	Q2TBR3	LOC782688			0.6276984	0.6372008
RAB35, member RAS oncogene family	A5D7E0	RAB35		1.6433071		1.6116684
Glucosidase 2 subunit beta	Q28034	PRKCSH	1.52164	1.7506983		
Bridging integrator 1	Q2KJ23	BIN1		1.5930014		1.6906537
Y-box-binding protein 1	P67808	YBX1		2.4457459	0.287731	
Cadherin-13	Q3B7N0	CDH13	1.9128225	2.9823395	1.5196257	2.3692946
Prolyl endopeptidase	Q9XTA2	PREP				0.5954978
Fibrinogen gamma-B chain	Q3SZZ9	FGG	1.5344916	1.8256537		
Histone H2A	Q32LA7	H2AFV				0.523695

Heterogeneous nuclear ribonucleoprotein U	Q08DH6	HNRNPU			0.5380552	0.6374487
Sodium/potassium-transporting ATPase subunit alpha	Q8HYW6	ATP1A3		1.8552683		2.1231766
Contactin-1	Q28106	CNTN1				1.7109184
AP-2 complex subunit alpha	Q0VCK5	AP2A1	0.5807257	0.5210476		
Fc-gamma-RII-D	A8DC37	FCGR2A	5.7022619	4.7644561		
Thyroglobulin	F1MPH3	TG	7.7323548		4.4865924	
Antithrombin-III	F1MSZ6	SERPINC1	1.5426078			
Peroxiredoxin 6	A0A3S8XGA0	Prdx6			1.9056353	
Septin-11	A2VE99	SEPTIN11		1.5130726		
GLOBIN domain-containing protein	A0A452DIQ5	HBA1	1.974396		2.1919656	
60S ribosomal protein L17	Q3T025	RPL17			0.5142695	
60S ribosomal protein L23a	Q24JY1	RPL23A		1.5150105	0.4819989	
SERPINE1 mRNA binding protein 1	Q2KJD1	SERBP1		2.1472469	0.4283982	0.6666995
40S ribosomal protein S26 (Fragment)	Q56JV1					0.5352079
Sodium/potassium-transporting ATPase subunit alpha-2	A2VDL6	ATP1A2		2.4929903		3.0723147
HNRNPR protein	A3KMV6	HNRNPR	1.7233523			
Histone H2B	A5A4L2	H2B	1.5645938			0.62942
Alpha-actinin-4	A5D7D1	ACTN4				0.6182985
Uncharacterized protein	A5D7Q2		3.1862436	1.6072902		
Lactoperoxidase	A5JUY8	LPO	0.5233992	0.2977981		0.6358619
SAFB protein	A5PJP2	SAFB	1.5011722		1.6927983	
ECM1 protein	A5PJT7	ECM1		1.768382		
40S ribosomal protein S7	A6H769	RPS7		1.9216625	0.5422079	
CBX3 protein	A6H7C4	CBX3		0.4451127		0.6091001
40S ribosomal protein S9	A6QLG5	RPS9			0.5247111	0.4737467
Adenosylhomocysteinase 3	A6QLP2	AHCYL2		3.2663551		7.9630689
AARS protein	A6QLT9	AARS1			0.5487062	
Uncharacterized protein	A6QM09		3.8017713	2.1841931		
NID1 protein	A6QNS6	NID1	1.581666			
SYN1 protein	A6QNW4	SYN1				1.8782474
CD5 molecule like	A6QNW7	CD5L	3.0814768	1.6296259		
SERPIND1 protein	A6QPP2	SERPIND1	2.06076			
Serpin A3-8	A6QPQ2	SERPINA3-8	2.7672639			0.5388472
Sulfhydryl oxidase	A6QQA8	QSOX1		0.6350933		0.6601175
Protein kinase C and casein kinase substrate in neurons protein 1	A7MBI0	PACSIN1		2.395819		3.3783765
Major allergen beta-lactoglobulin	B5B0D4		0.6283642			
Cumulus cell-specific fibronectin 1 transcript variant	B8Y9T0		2.112883			
Galectin-1	C0HJQ1	LGALS1				0.636557
Lactoferrin (Fragment)	P24627			0.5394608		0.5107927
Alpha-1 acid glycoprotein	D2U6V0	agp	1.9054002	2.5464439		
Ceruloplasmin (Fragment)	D2U6Z5	cp	1.7814006	2.1978329		
RNA-binding motif protein, X chromosome	D3JUI8	RBMX	1.6748961	1.6505403		
Proliferating cell nuclear antigen (Fragment)	D7PVJ0	PCNA		1.7676651	0.4962355	0.6362072
Uncharacterized protein	E1B805	LOC528040	1.9474611	1.8079431		
Exportin 1	E1BE98	XPO1			0.5960899	
Globin B1	E1BEL8	HBE1	2.056608		4.1880531	1.6103634
Uncharacterized protein	E1BFN5	LOC100336868	1.6351849			0.5878325
Karyopherin subunit beta 1	E1BFV0	KPNB1			0.5695721	0.6492539
Phospholipase A2 inhibitor and LY6/PLAUR domain containing	E1BFZ1	PINLYP	1.7001354	1.760526		
Melanotransferrin	E1BG25	MELTF	1.5327215			
Heterogeneous nuclear ribonucleoprotein L	E1BIB4	HNRNPL	1.535839	1.6478356	0.6577887	
Amine oxidase	Q29437	SAO	2.482795	1.6811464		
Serpin family G member 1	E1BMJ0	SERPING1		0.6176311		
Caveolae associated protein 1	E1BNE7	CAVIN1			2.269869	
Conglutinin 1 (Fragment)	E7DSS8	CGN1				0.6381251
Sodium phosphate solute carrier family 34 member 2	E9NST3	Slc34a2	0.6374725			
ATP-binding cassette sub-family G (WHITE) member 2	E9NV81	ABCG2		0.6132137		
Laminin subunit gamma 1	F1MD77	LAMC1	1.6636765			
DNA topoisomerase 2	F1MDU7	TOP2A	1.7713259	1.6106489		0.6578869
Neurofilament medium polypeptide	O77788	NEFM				1.7903619
Alpha-1,4 glucan phosphorylase	F1MJ28	PYGM	1.744617		1.90368	
Biotinidase	F1MJM4	BTD		1.5684335		
Calcium dependent secretion activator	Q1KZG5	CADPS		3.0740777		4.9462191
Laminin subunit beta 1	F1MNT4	LAMB1	0.5774752	0.4581752		

Kininogen-1	F1MNV5	KNG1	1.9430944			
Myosin heavy chain 9	F1MQ37	MYH9		0.5404066		0.4294784
Nucleophosmin	F1MTV9	NPM1			0.5267732	0.6567867
Splicing factor 3b subunit 1	F1MX61	SF3B1			0.6377187	
V-type proton ATPase subunit H	F1MZL6	ATP6V1H	0.5789102			2.1313242
N-ethylmaleimide sensitive factor, vesicle fusing ATPase	F1MZU2	NSF		1.7628911		
Filamin A	F1N169	FLNA	1.5930499			
Stathmin	F1N1C2				0.5325631	
Neural cell adhesion molecule 1	F1N1W7	NCAM1		1.8602154		2.5201439
Dihydrolipoyl dehydrogenase	F1N206	DLD				1.6698177
Thrombospondin-1	F1N3A1	THBS1		0.5005756		0.4321991
Collagen type IV alpha 5 chain	F1N474	COL4A5			1.6507565	1.6985863
60S ribosomal protein L5 (Fragment)	F4YCZ9	60SRPL5		1.6955829	0.5288629	0.6049209
Heat shock 90kDa protein 1 beta (Fragment)	F4YD16	H	1.738211	1.6716726		0.6653314
Ribosomal protein L23a (Fragment)	F4YD24	RPL23A		1.519236	0.4945854	
Ribosomal protein S19 (Fragment)	F6K453	rps19	2.0266524			0.5210191
Tumor rejection antigen 1 (Fragment)	F6K456		1.6177922			
Caveolae associated protein 2	F6PXE3	CAVIN2			3.5347502	2.9545471
Phosphoglucomutase 2	F6QEU6	PGM2				0.568889
Protein arginine methyltransferase 1	F6RFE5	PRMT1			0.5920864	
Neuroplastin	F6RM11	NPTN				2.1504941
Cdc42 protein	G1JRQ4			1.63674		
Gap junction protein	G3JXZ2	Cx43		2.062113		2.338066
Citrate synthase	G3JXZ3	CS		1.5784849		
DEAD-box helicase 3 X-linked	G3MWJ8	DDX3X		1.5380466		
60S ribosomal protein L31	Q56JX3				0.5996358	0.6329422
Coiled-coil domain containing 88A	G3MXG3	CCDC88A	2.6753888		3.0776802	1.8359889
Afamin	G3MYZ3	AFM	2.8615345		2.022322	0.6374859
Ig-like domain-containing protein	G3N148		2.7040695	1.5442977		
Uncharacterized protein	G3N342		2.105646	2.9131849		
Non-POU domain containing octamer binding	G3N361	NONO	1.5160891			
Heat shock protein HSP 90-beta	Q76LV1	HSP90AB1			0.636412	0.6077457
Transaldolase	G5E5C8	TALDO1	1.5122529			
Proteolipid protein (Fragment)	G8GUK4	PLP		2.5428002		3.3916728
Lipoprotein lipase	H9BTR2		0.4998609	0.299616		
Lipopolysaccharide binding protein	I0DHI6	LBP		0.615014		0.6614005
Mucin1-cell surface associated protein	I6X9J1			0.5747413		
Ubiquitin carboxyl-terminal hydrolase (Fragment)	I7FHM0			1.7588934		1.997109
HSP10	J9VCT4	H	1.6895375	1.6459926		
L-lactate dehydrogenase	K0IT60	LDHB				0.663241
CCCTC-binding factor	K7THR4	CTCF	1.9861775			0.6011301
Sodium/potassium-transporting ATPase subunit beta	L0CMU6			1.7055354		1.8939599
Haptoglobin	M1JVB9	HP	1.840737	2.7784827		
Myosin heavy chain 2x (Fragment)	M1Q746	MyHC2x	2.0816178		2.0444719	
Myosin heavy chain (Fragment)	M1R8X4	MyHC	1.7832913		2.4556596	
CD36	M9WLX8	CD36	0.5111116	0.5346946		
40S ribosomal protein S2	O18789	RPS2			0.5064457	0.5030328
Transthyretin	O46375	T	2.9538464	1.9920392		
Desmin	O62654	DES		0.203825	4.0981536	
Adenylate kinase isoenzyme 1	P00570	AK1	1.6913474	1.6918333	1.8836018	1.8841429
Prothrombin	P00735	F2	1.855188			
Collagen alpha-1(I) chain	P02453	COL1A1	3.2059385		3.6953138	
Neurofilament light polypeptide	P02548	NEFL		2.6648242		3.0043092
Fibrinogen alpha chain	P02672	FGA		1.7249085		
Fibrinogen beta chain	P02676	FGB		1.7054953		
Myelin basic protein	P02687	MBP		2.1787377		3.38598
Folate receptor alpha	P02702	FOLR1		0.4990353		
Beta-lactoglobulin	P02754	LGB	0.5165832	0.3479966		
Collagen alpha-1(III) chain	P04258	COL3A1				2.6721581
Protein disulfide-isomerase	P05307	P4HB				0.661123
Plasminogen	P06868	PLG	1.7221572			
Beta-1,4-galactosyltransferase 1	P08037	B4GALT1	0.4898765	0.390275		
Heterogeneous nuclear ribonucleoprotein A1	P09867	HNRNPA1		1.5924253	0.5014997	0.639542
Histone H2A type 1	P0C0S9			0.4158155		0.3281758
Glyceraldehyde-3-phosphate dehydrogenase	P10096	GAPDH		1.7031762		
High mobility group protein B1	P10103	HMGB1	1.7195779			0.5888072
Fatty acid-binding protein, heart	P10790	FABP3	0.3122906	0.3271148		
Phosphate carrier protein, mitochondrial	P12234	SLC25A3	0.6593165			

Alpha-2-HS-glycoprotein	P12763	AHSG	2.1323605			0.6582102
Glutamine synthetase	P15103	GLUL				1.5954482
Beta-2-glycoprotein 1	P17690	APOH	2.0948697	1.5038157		
Clusterin	P17697	CLU	1.5143345	2.369355	0.607767	
Gap junction alpha-1 protein	P18246	GJA1		2.2068267		2.8953637
Retinol-binding protein 4	P18902	RBP4	2.0518404			0.5860198
ATP synthase subunit alpha, mitochondrial	P19483	ATP5F1A		1.5110946		
Biglycan	P21809	BGN	2.9991766		2.3794394	
Platelet glycoprotein 4	P26201	CD36	0.3797763	0.3833554		
Prosaposin	P26779	PSAP	0.3360041	0.3614671		
Alpha-2-antiplasmin	P28800	SERPINF2	2.6173126	2.1806642		
Glutathione S-transferase P	P28801	GSTP1	1.9280869			
Alpha-1-antiproteinase	P34955	SERPINA1	2.0241291	1.6108566		
Neurogranin	P35722	NRGN		3.0392523		2.3837799
Protein disulfide-isomerase A3	P38657	PDIA3	2.2082205	1.5406552		
60S acidic ribosomal protein P2	P42899	RPLP2			0.4168556	0.5405906
Calreticulin	P52193	CALR				0.6111897
Syntaxin-1B	P61267	STX1B		1.7248803		2.3264955
60S ribosomal protein L27	P61356	RPL27			0.4648094	0.6444197
Syntaxin-binding protein 1	P61763	STXBP1		2.3609154		2.0618059
Myeloid-derived growth factor	P62248	MYDGF	1.5020769	1.9785235		
Hemoglobin subunit beta	P67820	HBB	0.5481866	1.6826444		2.2144308
Voltage-dependent anion-selective channel protein 2	P68002	VDAC2		1.6164855		
Elongation factor 1-alpha 1	P68103	EEF1A1	1.5349579			0.5560187
Caveolin-1	P79132	CAV1		1.6930397		
Brain acid soluble protein 1	P80724	BASP1		1.7558136		2.3169922
Complement factor B	P81187	CFB	1.6037408	1.608137		
Polymeric immunoglobulin receptor	P81265	PIGR				0.642271
Histone H3.2	P84227			0.5677193		0.4221262
Myoglobin	P84997	MB	3.0674503	0.5740973	3.5871426	
Cytochrome c oxidase subunit NDUFA4	Q01321	NDUFA4		2.4197852		2.1219282
Isocitrate dehydrogenase [NADP], mitochondrial	Q04467	IDH2	2.082236	0.4653325	2.6121008	0.5837453
Lumican	Q05443	LUM	3.0690244	2.2019176		
IGK protein	Q05B55	IGK	4.0003206	2.902892		
Alpha-internexin	Q08DH7	INA		1.8518645		2.1879914
MYL1 protein (Fragment)	Q08E10	MYL1	1.7801593		2.2478796	
Profilin-2	Q09430	PFN2				1.9535024
Odorant-binding protein-like	Q0IIA2	MGC151921	2.9855049	0.519498	1.9762003	0.3438722
MYOZ1 protein	Q0IIE1	MYOZ1			2.2120378	
Ras-related protein Rab-18	Q0IIG8	RAB18		0.6053911		
Isocitrate dehydrogenase 1 (Fragment)	Q0QEQ4	IDH1		0.3933476		0.527339
Succinate dehydrogenase (quinone) (Fragment)	Q0QF04	SDHA		1.8891104		
Tripeptidyl-peptidase 1	Q0V8B6	TPP1		0.5013092		
V-type proton ATPase subunit G	Q0VCV6	ATP6V1G2				4.1257773
Endoplasmic reticulum chaperone BiP	Q0VCX2	HSPA5	1.6837338			
Cellular repressor of E1A-stimulated genes 1	Q148D9	CREG1		0.5185666		
Membrane-associated progesterone receptor component 1	Q17QC0	PGRMC1	0.4810261	0.4560244		1.9203136
Beta-synuclein	P33567	SNCB		2.2105363		2.4023948
Histone H2A	Q17QG8	H2AFX		0.5696413		0.5057984
Synaptosomal-associated protein 25	Q17QQ3	SNAP25		1.7217909		1.7439048
Eukaryotic translation elongation factor 1 gamma (Fragment)	Q1JPA2	EEF1G	1.5649402			0.6136142
Mitotic checkpoint protein BUB3	Q1JQB2	BUB3			0.6216456	
Sodium-dependent phosphate transport protein 2B	Q27960	SLC34A2	0.5934376	0.6310936		
Sodium/potassium-transporting ATPase subunit beta-2	Q28030	ATP1B2		1.8418962		1.9222291
Alpha lactalbumin (Fragment)	Q28049	alfaLA	0.4735509	0.3449313		
Epithelial mucin (Fragment)	Q28078	MUC1		0.4891582	1.5185204	
Complement factor H	Q28085	CFH	1.7664157			
Serotransferrin	Q29443	TF	2.461642			0.6582412
Complement component C6	Q29RU4	C6	1.524502	1.6276789		
Leucine-rich alpha-2-glycoprotein 1	Q2KIF2	LRG1		1.8089571		
Tetranectin	Q2KIS7	CLEC3B	2.660019	3.5820021		
Protein HP-25 homolog 2	Q2KIU3		2.3192292	3.0452508		
EH-domain containing 2	Q2KJ47	EHD2	1.6272451	0.5292938	2.66324	
Cell division control protein 42 homolog	Q2KJ93	CDC42		1.635847		
Alpha-1B-glycoprotein	Q2KJF1	A1BG		1.5657409		
Serpin H1	Q2KJH6	SERPINH1	1.6882041		0.6329713	0.3537501

Septin-2	Q2NKY7	SEPTIN2	0.6147279			
60S ribosomal protein L7a	Q2TBQ5	RPL7A	1.8032309	2.1162071		
Testin	Q2YDE9	TES	2.3551457			0.3734496
T-complex protein 1 subunit alpha	Q32L40	TCP1				0.5811259
Histone H2B type 1-N	Q32L48	H2BC15				0.4825668
Myomesin (M-protein) 2, 165kDa	Q32LP3	MYOM2	2.6150025		4.3592198	
40S ribosomal protein S19	Q32PD5	RPS19	2.3519228	1.9360623	0.6434971	0.5297157
LIM and SH3 domain protein 1	Q3B7M5	LASP1	1.6646498			
Alpha-actinin-1	Q3B7N2	ACTN1	1.8387298			0.5121645
Beta-casein (Fragment)	Q3HW31	beta-casein		2.0673184		
Mitochondrial cytochrome c (Fragment)	Q3LUG8		1.6634825			
Claudin-11	Q3MHK4	CLDN11	1.5287506			
Vitamin D-binding protein	Q3MHN5	GC	1.8961473	1.6557975		
Immunoglobulin J chain	Q3SYR8	JCHAIN	2.3030183	1.639749		
Elongation factor 2	Q3SYU2	EEF2			0.5587146	0.6181222
HNRPM protein	Q3SYX2	HNRPM				0.5860804
Transcription elongation factor B (SIII), polypeptide 2 (18kDa, elongin B)	Q3SZ32	TCEB2				0.6170689
Eukaryotic initiation factor 4A-I	Q3SZ54	EIF4A1			0.5539319	0.6081564
Phosphoglycerate mutase 1	Q3SZ62	PGAM1				0.6652797
Enhancer of rudimentary homolog	Q3SZC0	ERH			0.5646107	0.5523438
Lamin A/C	Q3SZI2	LMNA	1.7757738			
60S ribosomal protein L32	Q3SZQ6	RPL32				0.5543762
Hemopexin	Q3SZV7	HPX	2.4555231	2.5777548		
Carbonic anhydrase 3	Q3SZX4	CA3			1.7626211	
Serum amyloid P-component	Q3T004	APCS	2.7616035	2.520618		
60S ribosomal protein L30	Q3T0D5	RPL30				0.6498855
40S ribosomal protein S10	Q3T0F4	RPS10				0.5997696
Calumenin	Q3T0K1	CALU		0.6535962		0.4802979
T-complex protein 1 subunit gamma	Q3T0K2	CCT3	2.0525902	1.7822753		0.5621356
60S ribosomal protein L8	Q3T0S6	RPL8		1.6040216		
40S ribosomal protein S11	Q3T0V4	RPS11				0.5584161
WAP four-disulfide core domain 2	Q3T0Z0	WFDC2		0.5663393		
Heat shock protein beta-1	Q3T149	HSPB1	2.2002422	1.6186292		
Four and a half LIM domains 1	Q3T173	FHL1	1.8634663		3.6629739	
40S ribosomal protein S23	Q3T199	RPS23	2.0506013			
Glucose-6-phosphate isomerase	Q3ZBD7	GPI	1.8287807	1.5049412		
Proteasome subunit alpha type-7	Q3ZBG0	PSMA7			0.5723619	0.6429353
Proliferation-associated 2G4, 38kDa	Q3ZBH5	PA2G4				0.6248123
PDZ and LIM domain 5	Q3ZBU0	PDLIM5	1.5785543			
Tubulin beta-4A chain	Q3ZBU7	TUBB4A				2.0719116
Fructose-bisphosphate aldolase	Q3ZBY4	ALDOC		0.5016947		
Beta-enolase	Q3ZC09	ENO3	1.8955196		2.3873615	
Heterogeneous nuclear ribonucleoprotein A/B	Q3ZC44	HNRNPAB	1.6693215	3.5046757	0.2518419	0.5287323
Proteasome subunit alpha type-4	Q3ZCK9	PSMA4				0.6140675
Cysteine-rich secretory protein 2	Q3ZCL0	CRISP3	0.411343	0.5591854		
Broad substrate specificity ATP-binding cassette transporter ABCG2	Q4GZT4	ABCG2		0.6379551		
Glyceraldehyde-phosphate dehydrogenase (Fragment)	Q56AP4	GAPDH				1.9994619
40S ribosomal protein S3a	Q56JV9	RPS3A		1.536206	0.6207985	
X-linked eukaryotic translation initiation factor 1A	Q56JW9				0.5713002	0.591316
40S ribosomal protein S28	Q56JX6	RPS28			0.4162978	0.5479548
60S ribosomal protein L27a	G1K1B4	RPL27A	1.6403522	1.843602	0.4383903	0.4927096
Cysteine-rich protein 1	Q56K04	CRIP1	1.8587254			
Fetuin-B	Q58D62	FETUB	2.4715051			0.4937578
Solute carrier family 3 (Activators of dibasic and neutral amino acid transport), member 2	Q58DQ6	SLC3A2	2.7475002	1.9684984		
60S ribosomal protein L7	Q58DT1	RPL7			0.6214499	
DnaJ homolog subfamily A member 1	Q5E954	DNAJA1	1.6498278			0.6207844
Triosephosphate isomerase	Q5E956	TPI1	1.57698			
40S ribosomal protein S8	Q5E958	RPS8			0.5111052	0.6420472
Proteasome subunit alpha type-5	Q5E987	PSMA5	1.7698808	1.6466719		
60S ribosomal protein L10a	Q5E9E6	RPL10A				0.552795
RNA-splicing ligase RtcB homolog	Q5E9T9	RTCB	1.5853934			
Aldose 1-epimerase	Q5EA79	GALM				0.6088727
D-3-phosphoglycerate dehydrogenase	Q5EAD2	PHGDH			0.6367111	
Alpha-1-acid glycoprotein	Q5GN72	agp	2.0540638	3.2302903		
Tropomyosin beta chain	Q5KR48	TPM2			2.329557	
Cathelicidin (Fragment)	Q5XL27	Cath	1.7558543			
Fatty acid-binding protein 3	Q5XLB1	FABP3	0.4110799	0.4047817		
Eukaryotic translation initiation factor 5A-	Q6EWQ7	EIF5A		1.5669415	0.507366	0.6064278

1						
3-hydroxy-3-methylglutaryl-coenzyme A synthase 1 (Fragment)	Q3ZC79	HMGCS1		1.7466632	0.4529587	
Growth/differentiation factor 8	Q6X5V1	MSTN	2.1594133	1.8721086		
40S ribosomal protein S15a	Q76I82	RPS15A	0.6533268	0.4826911		
Alpha-2-macroglobulin	Q7SIH1	A2M	2.2084186	2.0300716		
Reticulon	F1N405	RTN4		0.656411	0.2972278	0.1622384
Chitinase-3-like protein 1	Q7YS85	CHI3L1				0.5660571
Similar to ribosomal protein L3 (Fragment)	Q862D2			1.7217022	0.5786668	
Ferritin (Fragment)	Q862D5			0.6075597		0.6206607
Similar to S3 ribosomal protein (Fragment)	Q862D8			1.8099088	0.5650975	
Similar to ribosomal protein L26 (Fragment)	Q862N4					0.5780296
Similar to ribosomal protein L18a (Fragment)	Q862R3				0.5732279	0.5688133
Mucin-15	Q8MI01	MUC15	0.6099179			
PC4 and SFRS1-interacting protein	Q8MJG1	PSIP1	1.6829529		0.419325	0.3400247
Troponin T fast skeletal muscle type	Q8MKH8	Tnnt3			2.8583745	
Legumain	Q95M12	LGMN	0.5643782	0.4046929		
Endoplasmin	Q95M18	HSP90B1	1.6373109			
Myosin-7	Q9BE39	MYH7			2.053552	
Myosin-2	Q9BE41	MYH2	1.6200257		2.0880334	
Dolichyl-diphosphooligosaccharide--protein glycosyltransferase 48 kDa subunit	Q9TS10			0.3301656		0.2364173
Alpha-lactalbumin	Q9TSN6	LALBA	0.4804972	0.3708023		
Hemoglobin subunit alpha-1	Q9TSN7			2.2555425		
Hemoglobin subunit alpha-2	Q9TSN8					1.5530935
Chloride intracellular channel protein 4	Q9XSA7	CLIC4		1.6962449		
Creatine kinase M-type	Q9XSC6	CKM	1.98589		2.3905767	
Serum amyloid A protein	V5LEH6	SAA3		2.3741696	0.5441508	
Transgelin	Q9TS87	TAGLN	2.66651		1.6881663	
Heat shock protein A1A	W6CNS2		1.67169	1.5578215		

* Fold change at p-Value < 0.01.
